# Multiomics Evaluation of Human Fat-Derived Mesenchymal Stem Cells on an Osteobiologic Nanocomposite

**DOI:** 10.1089/biores.2020.0005

**Published:** 2020-02-21

**Authors:** Austin Bow, Bailey Jackson, Christopher Griffin, Sara Howard, Hector Castro, Shawn Campagna, Alexandru S. Biris, David E. Anderson, Shawn Bourdo, Madhu Dhar

**Affiliations:** ^1^ Department of Large Animal Clinical Sciences, College of Veterinary Medicine, University of Tennessee, Knoxville, Tennessee.; ^2^ Center for Integrative Nanotechnology Sciences, University of Arkansas at Little Rock, Little Rock, Arkansas.; ^3^ Biological and Small Molecule Mass Spectrometry Core and the Department of Chemistry, University of Tennessee, Knoxville, Tennessee.

**Keywords:** multiomics, metabolomics, transcriptomics, scaffold, mesenchymal stem cells

## Abstract

Effective graft technologies for bone repair have been a primary focus in the field of bone tissue engineering. We have previously fabricated and examined a nanocomposite composed of polyurethane, nano-hydroxyapatite, and decellularized bone particles, which demonstrated osteobiologic characteristics. To evaluate the underlying mechanisms of this biomaterial, human adipose-derived mesenchymal stem cell seeded scaffolds were assessed using a combinatorial approach of transcriptomic and metabolomic analyses. Data from osteogenic and signal transduction polymerase chain reaction arrays and small molecule abundances, measured through liquid chromatography–mass spectrometry, were cross-examined using Integrated Molecular Pathway Level Analysis, Database for Annotation, Visualization, and Integrated Discovery, and ConsensusPathDB online tools to generate a fundamental collection of scaffold-influenced pathways. Results demonstrated upregulation of key osteogenic, cellular adhesion cell signaling markers and indicated that Hedgehog and Wnt signaling pathways were primary candidates for the osteobiologic mechanisms of the scaffold design. The detection of complimentary metabolites, such as ascorbate, further indicates that scaffolds generate intricate cellular environments, promoting cell attachment and subsequent osteodifferentiation.

## Introduction

The field of bone tissue engineering faces unique challenges in biomaterial design stemming largely from the highly dynamic nature of the target tissue. Native bone undergoes continuous remodeling through osteoblastic (OB) and osteoclastic activity to accommodate for mechanical forces exerted on the body and provide structural support. For this reason, noncompromised bone tissue, as opposed to that observed in osteoporotic or geriatric individuals, is innately capable of repairing sizable injuries. However, for cases of tissue damage that result in defect sizes that exceed the reparative capacities of native bone, or for accelerated repair, the application of a graft material is necessary.^1^

Currently the gold standard for such graft material is the use of autologous tissue, as this eliminates concerns of immunogenic reaction and provides an optimal substrate for cellular on-growth and eventual integration. Despite the superior reparative and restorative functions of autografts, implementation incurs an increased risk to patient due to the need for multiple surgical sites and donor site morbidity, as well as a reliance on a limited source material.^2^ Therefore, the development and characterization of graft materials with similar or enhanced functionality and biocompatibility to autografts offer an attractive alternative.^2–4^

Scaffold constructs being designed for bone tissue engineering must display key osteobiologic characteristics, including osteoinductive, osteoconductive, and osseointegrative functions, to facilitate effective repair of native tissue.^5–8^ Osteoinduction indicates that the material is capable of stimulating exposed cells toward an osteogenic lineage.^2^ The osteoconductivity of a material determines the ability of cellular communication across and through a substrate.^2^ Finally, osseointegration indicates the measure of cell migration and subsequent formation of mature bone tissue on the surface and throughout the matrix.

We have previously reported the fabrication of a nanocomposite composed of nano-hydroxyapatite (nHA)/polyurethane (PU) film layers with interspersing layers of decellularized bovine bone particles (DBPs), which demonstrated biocompatibility and osteobiologic characteristics, both *in vitro* and *in vivo*.^4,9^ Specifically, 8-week-old Sprague Dawley rats had a significant increase in new bone formation over a 30-day period within unicortical tibial bone defects when treated with the nanocomposite. Based on these results, we next wanted to elucidate more precise mechanisms by which exposed cells are influenced. To accomplish this, a multiomics approach utilizing analytical tools of transcriptomics and metabolomics was implemented.^3^

The use of various molecular analytical tools to assess the functions of biomaterials is an expanding and promising approach as the critical attributes of bone scaffolds previously described depend heavily on the cell–biomaterial interactions.^7^ Stimuli from surface topography or composition elements can drastically alter the influence of a material on exposed cells, leading to substantially different results both *in vitro* and *in vivo*.^9^ Transcriptomics, the study of messenger RNA (mRNA) molecules and functional impact of their expression levels, offers the potential to observe the fundamental regulative capacities of cells through comparative assessment of gene expression.^10^

The extraction and analysis of mRNA from cells exposed to various conditions, through generation of complimentary DNA (cDNA) and subsequent real-time quantitative polymerase chain reaction (PCR), offer the potential to evaluate the effect of specific treatments on exposed cells by normalizing to an untreated control culture. Such methods have been utilized in studies focused on elucidating correlations between discrete material characteristics and biological responses of exposed cells in attempts to establish pathway libraries. These data can be used as a rationale to design biomaterials with specific topographies, architecture, and composition.^3^

Transcriptomic evaluation is further strengthened through supplementation with metabolomic data, which consist of small molecule concentrations often detected through mass spectrometry.^11^ Metabolites and their associated relative abundance can be detected in a wide variety of samples ranging from acellular materials to tissue biopsy samples, providing the potential for comparative analyses based on metabolite profiles.

By cross-examining detected small molecules with expression data for upregulated and downregulated genes, respectively, it is possible to develop a basic pathway(s) to describe the behavior of cells on scaffolds. Online databases, such as the Kyoto Encyclopedia of Genes and Genomes (KEGG) and Reactome, and tools for assessing connective elements within datasets, including Integrated Molecular Pathway Level Analysis (IMPaLA), Database for Annotation, Visualization, and Integrated Discovery (DAVID), and ConsensusPathDB (CPDB), can be used to generate basic pathway maps demonstrating the signals that are triggered when cells interact with scaffolds.

The use of naive cells during *in vitro* examination of scaffold mechanisms provides a more relevant model, as predifferentiated and immortalized cell lines may demonstrate expression profiles that reflect innate cell programming instead of material induced effects. Naive cells alleviate this concern and permit accurate assessment of material impact on cellular activity. Furthermore, the use of human mesenchymal stem cells (hMSCs) contributes a clinical translatability aspect, as the designed scaffold technology is intended for human medicine. Therefore, studies conducted to elucidate mechanisms of the biomaterial were facilitated using MSCs derived from human adipose tissue.

MSCs are naive multipotent cells with the potential to differentiate toward multiple lineages, namely osteocytes, chondrocytes, and adipocytes.^12^ These cells therefore offer a unique potential as a reparative element, especially when coupled with a scaffold substrate, and have been implemented in a wide array of cell-based treatments.^1^ Seeding of these adipose-derived hMSCs (adhMSCs) onto the nanocomposite scaffold can thereby evaluate the effectiveness of the construct as an osteogenic platform capable of application as a cell-based therapy device.^1^

Our objective in the present study, based on data from previous studies, is to assess the interaction between hMSCs and the nanocomposite through variations in both the transcriptional and metabolite landscapes. Expression of genes associated with osteogenesis, cellular attachment, and signaling was examined for hMSC seeded nanocomposites and compared with hMSCs differentiated through a well-established method to assess scaffold impact. These data were then cross-examined with small molecule concentrations to elucidate potential candidate pathways of effect for the scaffold on exposed cells.

## Materials and Methods

### Adipose-derived human MSCs

#### Cell collection, isolation, expansion, and characterization

adhMSCs were collected under an established IRB protocol, and primary cultures were developed using previously described methods.^12^ The stromal vascular fraction of cells, which contain the nonhematopoietic mesenchymal stem cells, were seeded in Dulbecco's modified Eagle's medium-F12 (DMEM-F12) growth media containing 10% fetal bovine serum and 1% amphotericin penicillin streptomycin and expanded *in vitro* in tissue culture polystyrene flasks.

Cells incubated at 37°C and 5% CO_2_, with growth media replaced every 2–3 days, were enzymatically released from substrates with 0.05% Trypsin-EDTA upon reaching ∼80% confluency and then allocated to tissue culture flasks for continued expansion, cryopreservation, or experimental setups. Cells were characterized and confirmed to be mesenchymal stem cells using previously described *in vitro* assays, including trilineage differentiation.^12^

### hMSC/scaffold constructs

#### Cell seeding

1 × 5 × 5 mm pieces of the nanocomposite scaffold material were cut from bulk scaffold blocks to ensure that each piece fits into a single well of a 24-well plate.^4^ Each scaffold piece was placed into individual wells of a nontissue culture plate, and cell solutions were directly added to ensure cellular migration into nanocomposite pores through capillary action.

Cells were seeded at a density of 4 × 10^4^ cells/scaffold for proliferation assessment and 5 × 10^5^ cells/scaffold for both gene expression and small molecule analyses. As previously observed, total RNA from cell/scaffold complexes at 5 days that had received media with osteogenic-inducing agents (growth media supplemented with 10 mM beta glycerophosphate, 10 nM dexamethasone, and 155 μM ascorbic acid) showed consistently poor yields.^4^ This indicated that stress factors due to conditions may negatively impact cell health, which was observed in previous studies.^4^ As such, cell seeded scaffolds were exposed to growth media lacking osteodifferentiation additives. Media was refreshed every 2–3 days.

#### Cell proliferation

Quantitative analysis of Calcein-AM fluorescent staining was performed as previously described to determine cellular proliferation and viability on the scaffolds.^9^ Briefly, cell-seeded scaffolds were cultured in black-walled 24-well plates, preventing light refraction across wells during reading, and assessed in triplicate at 3, 5, and 7 days of growth. Samples were incubated in 0.5 mL staining solution, containing 2 μg/mL calcein-AM dimethyl sulfoxide mix in HBSS, at 37°C for 5 min. Fluorescence intensity was quantitated using a plate reader setup with an excitation wavelength of 485 nm and an emission wavelength of 528 nm. Normalized average fluorescent intensity values from each time point were plotted to generate a cellular proliferation curve.

### Transcriptomics

#### Osteogenic and signal transduction gene expression

Total RNA was extracted from the cell/scaffold constructs 5 days postseeding as described previously and was analyzed for the expression of genes relating to osteogenesis and signal transduction.^4,10^ Total RNA isolation was performed with TRIzol extraction agent (Thermo Fisher Scientific, Waltham, MA) as per the manufacturer's protocol with modifications to increase the yield of RNA.^13^ cDNA was prepared using a Qiagen First Strand cDNA Reverse Transcription Kit (Qiagen). A housekeeping RT^2^ Profiler array (Qiagen) was used to ensure the quality of isolated RNA.

Expression was then evaluated using Qiagen RT^2^ Profiler arrays for human osteogenesis (PAHS-026Z) and signal transduction (PAHS-014Z) with 2 μg of total RNA per array with ∼20.8 ng cDNA per PCR, with samples run in triplicate. Relative fold differences in the gene expression and corresponding significance values were generated through Qiagen data center. Expression of cells seeded on scaffold constructs for 5 days was compared to cell monolayers differentiated on polystyrene substrates for 21 days with osteodifferentiation media.^9^

### Metabolomics

#### Small molecule analysis

Metabolite relative abundance profiles for cells exposed to scaffolds were compared with cell- and material-based controls to determine variations among groups. hMSCs seeded on material scaffolds for 5 days were compared to cell monolayers cultured on polystyrene substrates with and without osteodifferentiation media additives to evaluate concentration differences associated with the examined nanocomposite. In addition, acellular scaffold samples both exposed and not exposed to growth media were implemented to address metabolites attributed to the basal scaffold or media additives.

Samples, in triplicate, were collected by scraping wells, adding HBSS, and pelleting suspended samples. Pellets were isolated and weighed before storage at −80°C with cell monolayer, dry material blanks, media-exposed material blanks, and cell-seeded material samples having weight ranges of 16.53–25.64 mg, 30.14–50.37 mg, 92.08–175.82 mg, and 165.30–191.71 mg, respectively. Once all the samples were collected, the metabolites were extracted using a 20:40:40 solution of water/methanol/acetonitrile with 0.1 M formic acid following the procedure previously reported by Lu et al.^14^

Samples were reconstituted in ultrapure water and then ran on an LC-MS; they were separated on a Phenomenex Synergi Hydro RP column (100 × 2.0 mm, 2.5 μm pore size; Phenomenex, Torrance, CA) using ultra high-pressure liquid chromatography. The mobile phases used to elute the metabolites were (A) 97:3 water/methanol with 11 mM tributylamine and 15 mM acetic acid and (B) methanol. The 26-min gradient, adapted from Lu et al., was used with a flow rate of 0.2 mL/min. The gradient was as follows: 0 min, 0% B; 5 min, 20% B; 13 min, 55% B; 15.5 min, 95% B; 19 min, 0% B; and 25 min, 0% B.

The Exactive Plus Orbitrap Mass Spectrometer (Thermo Fisher Scientific) was operated in negative mode with an electrospray ionization probe; the scan range was set from 72 to 1,200 m/z while resolution was set to 140,000; the capillary temperature was set to 300°C.^15^ Once the raw files were obtained from the MS, the files were converted to mzML using an open source file converter. The mzML files were loaded into Metabolomic Analysis and Visualization Engine where metabolites were chosen based on peak shape and signal-to-noise ratio. Metabolite intensities were recorded and normalized to sample weight.

Appropriately formatted data were uploaded to MetaboAnalyst, an online metabolomic statistical analysis tool, to evaluate statistically significant variations among sample groups and generate visual representative figures. Sparse partial least squares discriminant analysis (sPLS-DA) performed on sample groups demonstrated similarity within groups and significant variation between groups. *Post hoc* heatmaps offer a visual representation of metabolite variations among study groups. Online databases and tools were then used to correlate detected metabolites and relative concentrations to potential pathways.

### Pathway analysis

#### IMPaLA and DAVID pathway assessment

Evaluation of molecular impact of the scaffold material on exposed cells was conducted through use of both IMPaLA and the DAVID online software tools, with gene and metabolite inputs entered using Entrez IDs and KEGG IDs, respectively.

For IMPaLA assessment, the detected metabolites were cross-examined with the upregulated genes identified in osteogenic and signal transduction arrays. The generated list of pathways consists of pathway names, database source for pathway information, target genes involved in pathway, and target metabolites involved in pathway. Pathways of interest (PoIs) were selected based on the number of target genes/metabolites included, with those pathways containing greater numbers of targets indicating more relevance to material impact.

DAVID assessment was then performed for pathway enrichment based on functional categories, gene ontology, pathways, protein domains, and tissue expression characteristics based on the default statistical parameters.^16,17^ Resulting PoIs are then divided into clusters based on pathway enrichment significance.

#### Network mapping

Visualization of pathway connections for both gene and metabolite elements was facilitated through the use of CPDB. Network map construction using CPDB was used to illustrate intra-omic connection based on gene expression and metabolite concentration data, respectively.

## Results

### Cell viability and proliferation

**FIG. 1.**
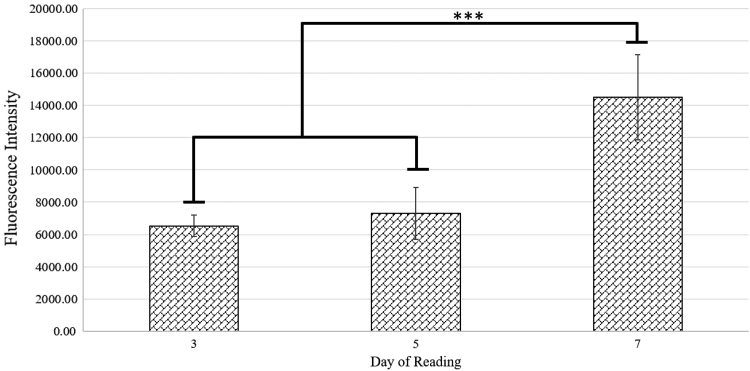
Calcein-AM proliferation assay conducted at days 3, 5, and 7 time points. Fluorescent intensity measurement output by plate reader is normalized to blank scaffold readings. The significant increase in fluorescence intensity between day 7 readings and previous time points is indicated by asterisks.

Calcein-AM staining demonstrated an increased fluorescent intensity in cell-seeded scaffolds at day 7 that was significantly >days 3 and 5 (Fig. 1). This increase over time indicates that scaffolds are cytocompatible.

### Transcriptomics

Established techniques using cells cultured for 21 days with osteodifferentiation media were used as a positive control to assess cells seeded onto scaffolds and cultured for 5 days. RNA expression was compared using PCR evaluation of RNA, extracted and isolated from these cell cultures, and was carried out utilizing Qiagen RT^2^ Profiler arrays for human osteogenesis and signal transduction pathways. Resulting expression fold changes and significance data between genes for samples were generated through Qiagen data center.

**FIG. 2.**
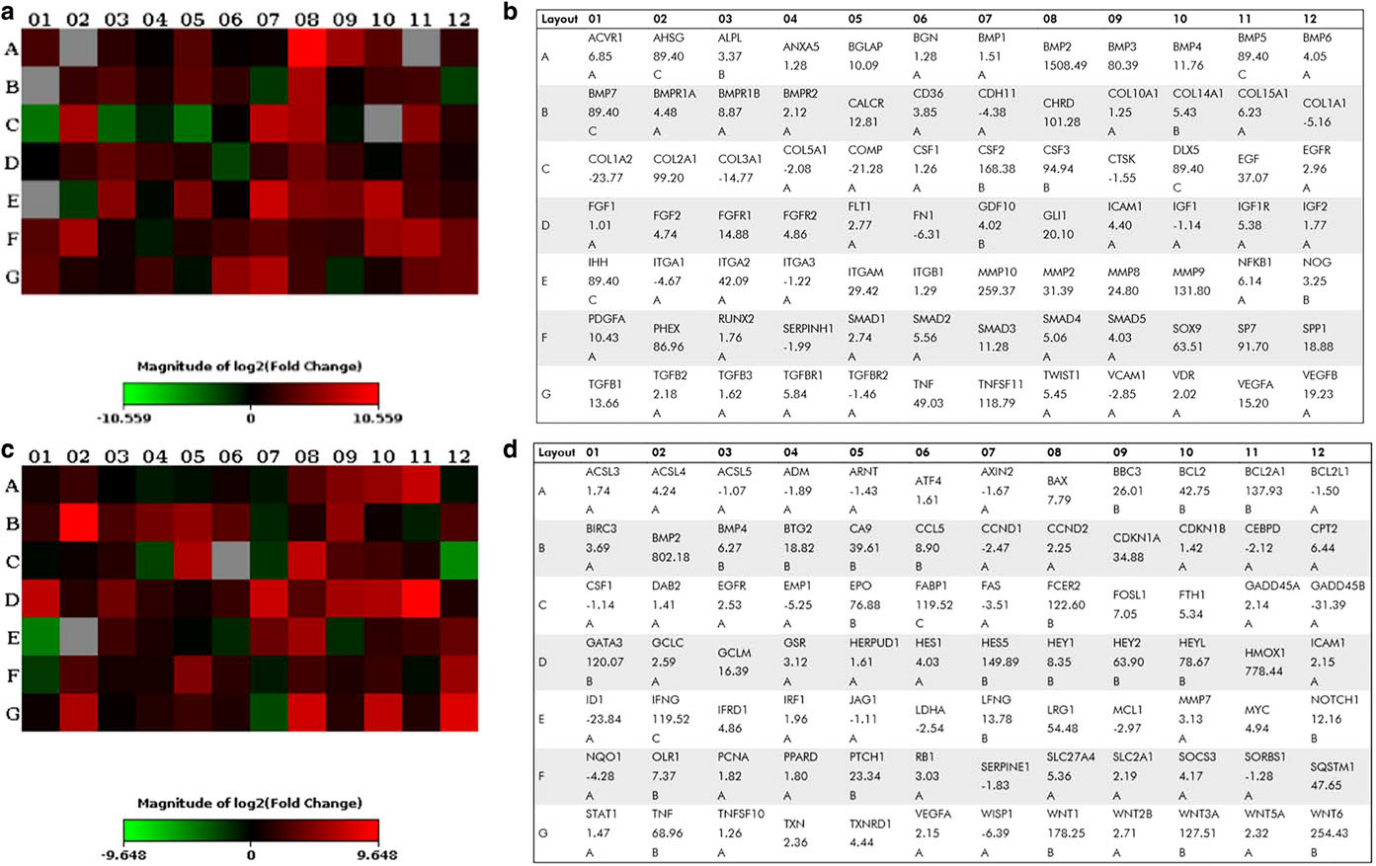
**(a, c)** Heatmap and **(b, d)** gene primer with expression values generated from human osteogenesis **(a, b)** and signal transduction arrays **(c, d)**. Heatmaps utilize a Log2 scale and represent expression fold changes in cell-seeded materials relative to differentiated cell monolayers. Complimentary gene primer lists display numerical expression fold change values for heatmaps with associated rankings shown directly below values. Rankings are assigned using Qiagen Data Center processing and indicate the quality of the expression relationship based on cycle threshold values of polymerase chain reaction runs. Primary attention was given to unranked and rank “A” genes, as these were most reliable values.

Fold change values and representative heatmaps for each array type are presented in Figure 2. Significantly upregulated genes, as designated by Qiagen Data Center output for genes with expression fold differences >2, and their associated functions can be viewed in Supplementary Tables S1–S4. Notably, expression of RUNX2, an essential gene in regulating bone formation and remodeling, did not significantly differ from cell controls stimulated through well-characterized differentiation agents toward osteolineage. Furthermore, data from signal transduction arrays showed enhanced expression of genes related to oxidative stress, Notch, Hedgehog (HH), and hypoxia signaling pathways.

### Metabolomics

Assessment of small molecule concentration profiles utilizing sPLS-DA showed that all sample groups were discrete and unique, with intragroup samples forming tight clusters. When comparing all study groups, it was determined that 18 metabolites were significantly responsible for driving the separation of sample groups. However, separation of study groups into standard cell culture (hMSC monolayers with and without osteodifferentiation media) and cell-free material (acellular scaffolds exposed and unexposed to growth media) subsets permitted more relevant comparisons due to initial sample characteristics, primarily weight and culture size. Subset groups both included hMSC-seeded scaffold samples.

**FIG. 3.**
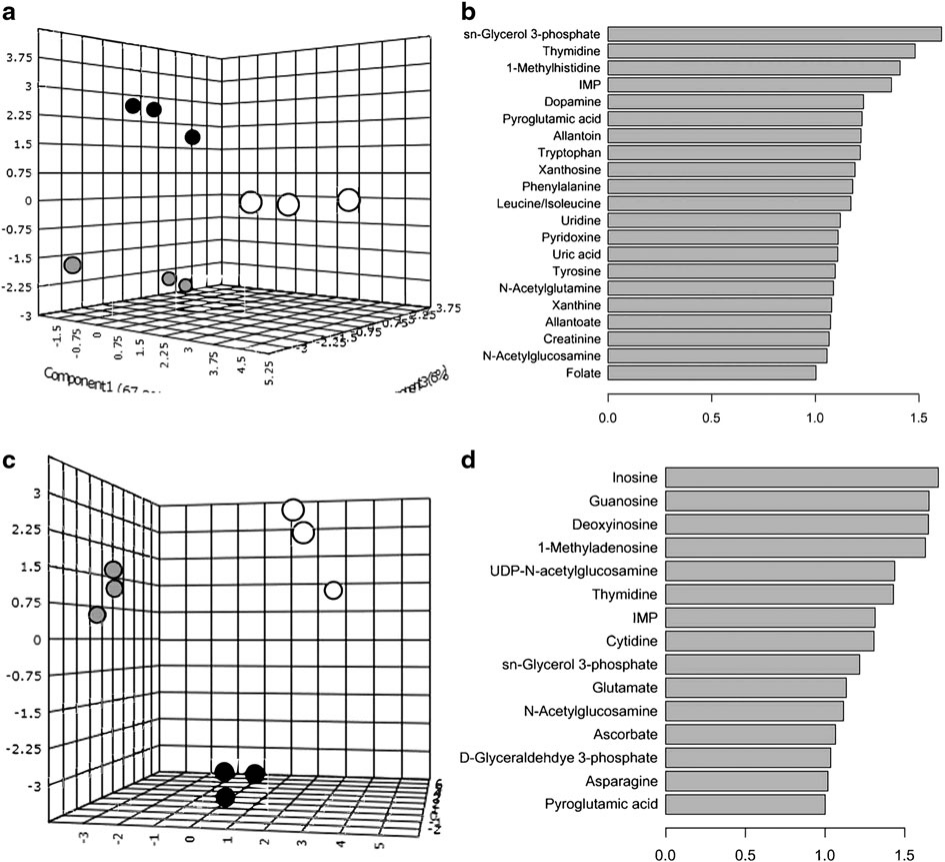
**(a)** sPLS-DA plot demonstrating discrete cluster separation for metabolomic concentration assessment of cell-based subset group with differentiated (gray) and undifferentiated (black) hMSCs on tissue culture substrates compared to hMSC-seeded (white) scaffolds. **(b)** Twenty-one metabolites driving separation observed in sPLS-DA plot. **(c)** sPLS-DA plot demonstrating discrete cluster separation for metabolomic concentration assessment of material-based subset group with dry (white) and media-exposed (gray) scaffolds compared to hMSC-seeded (black) scaffolds. **(d)** Fifteen metabolites driving separation observed in sPLS-DA plot. hMSC, human mesenchymal stem cell; sPLS-DA, sparse partial least squares discriminant analysis.

**FIG. 4.**
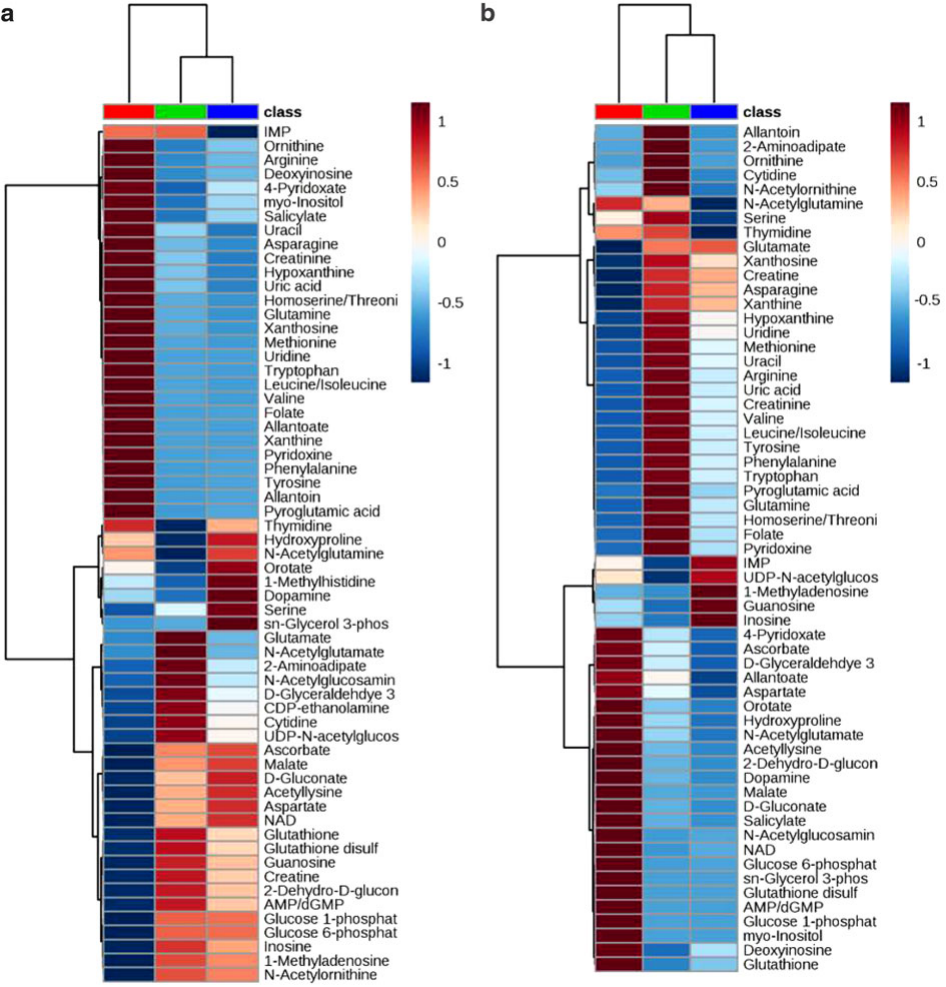
Heatmaps depicting relative abundance of metabolites among both **(a)** cell-based, with differentiated (green) and undifferentiated (blue) hMSCs on tissue culture substrates compared to hMSC-seeded (red) scaffolds, and **(b)** material-based subset groups, with dry (red) and media-exposed (green) scaffolds compared to hMSC-seeded (blue) scaffolds. Log scale used for relative abundance is Log2.

Although groups remained discrete and unique when examined with sPLS-DA, it was observed that the metabolites responsible for the distinct grouping varied in both number and significance with 21 metabolites for cell oriented and 15 metabolites for material-oriented samples (Fig. 3). Generation of corresponding heatmaps for cell-oriented and material-oriented groups was performed by normalizing hMSC-seeded scaffolds to subgroup-specific controls, undifferentiated hMSC monolayers, and acellular scaffolds unexposed to growth media (Fig. 4). The resulting heatmaps were assessed for metabolites of interest (MoIs), compounds that may serve as corollary links to cellular and material mechanisms.

For cell-oriented subset groups, comparisons in which both cell monolayers exposed to differentiation agents and cells seeded to scaffolds exhibited higher concentration than control samples, as well as those in which cell-seeded scaffolds alone displayed superior concentration, were selected as MoIs. The first of these relations being potentially indicative of an osteodifferentiation-related metabolite and the second representing a scaffold-related metabolite.

Similarly, MoIs from material-oriented subset groups were selected if a metabolite concentration was significantly different in cell-seeded scaffold samples compared to one or both control groups to examine changes due to cellular activity on constructs. Metabolites that demonstrated an increase in concentration in scaffold samples exposed to media compared to dry scaffold samples were considered to be associated with components of media and were therefore not further examined.

### Pathway analysis

#### IMPaLA and DAVID pathway assessment

PoI collections generated through IMPaLA software can be observed in Supplementary Table S5. Highly ranked PoIs for samples were largely associated with metabolic and signaling functions, including extracellular matrix (ECM) organization and cell differentiation pathways. PoIs selected from cluster lists, created utilizing DAVID, are displayed in Supplementary Table S6 with pathway enrichment score, number of overlapping genes, and significance values.

These generated PoIs demonstrate significant impact on bone mineralization, osteoblast differentiation, and osteoclast differentiation, which supports the osteogenic potential of the scaffold. Furthermore, cell signaling pathways, such as bone morphogenetic protein (BMP) signaling and cell–cell junction organization, compliment IMPaLA output and suggest that scaffolds facilitate cellular attachment and communication in addition to osteobiologic functions.

#### Network mapping

**FIG. 5.**
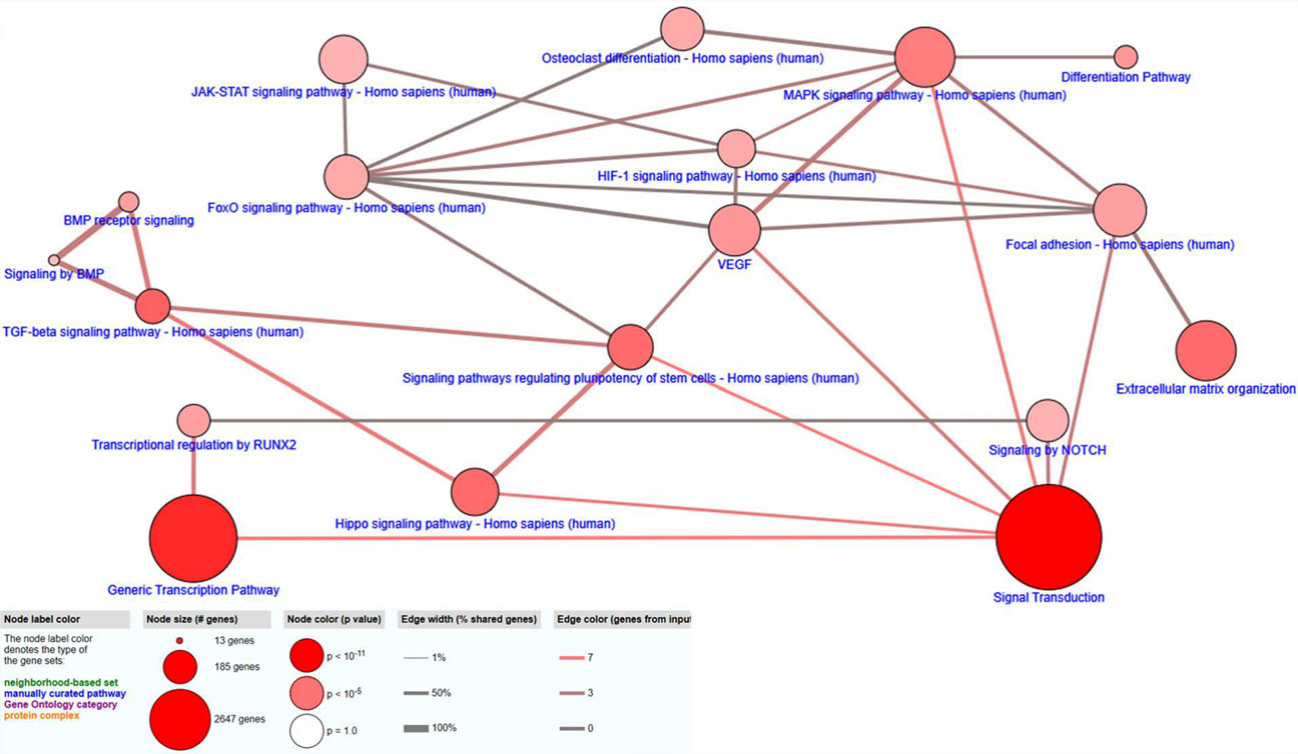
CPDB network map generated from upregulated osteogenesis and signal transduction array expression data with pathway elements selected from generated list based on significance (*p*-value <0.01) and relevance to utilized arrays. Pathway connective elements were filtered for at least a 0.15 overlap and 2 overlapping agents. CPDB, ConsensusPathDB.

**FIG. 6.**
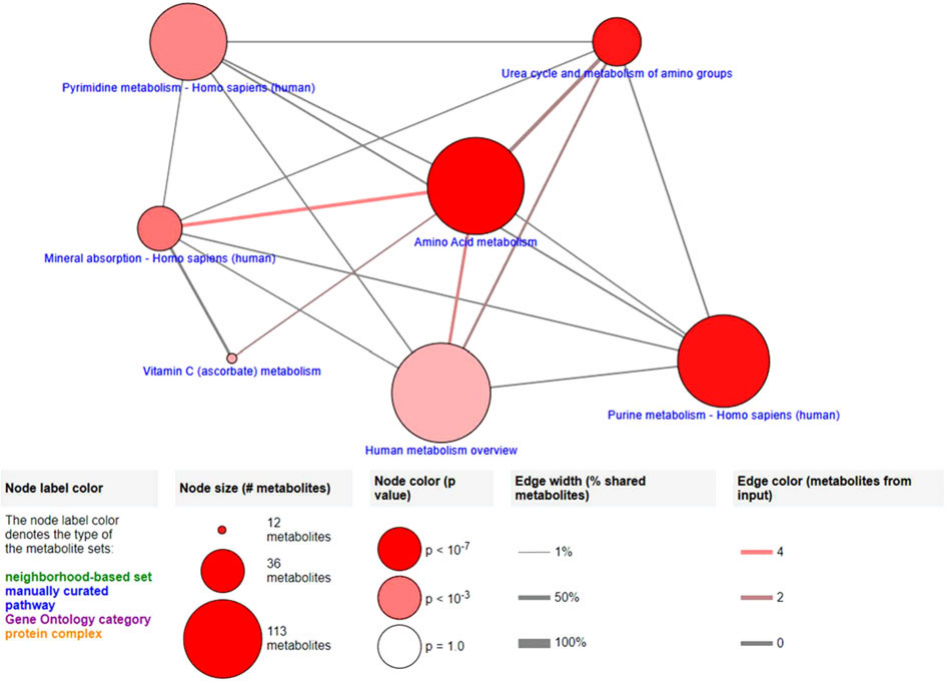
CPDB network map generated from metabolite concentration data with pathway elements selected from overview based on significance (*p*-value <0.01).

Observational assessment of intra-omic networks for gene expression and metabolite concentration data individually through CPDB demonstrated elements associated with osteogenic and cellular attachment functions (Figs. 5 and 6). As in IMPaLA and DAVID assessments, CPDB generated for transcriptomic and metabolomic data demonstrates pathways closely associated with osteogenesis and cellular attachment. Mapped transcriptomic data (Fig. 5) include pathways for cell differentiation, BMP signaling, and osteoclast differentiation, as well as ECM organization and focal adhesion pathways, and are reinforced by detection of fundamental metabolism functions and mineral absorption pathways through small molecule assessment with CPDB.

## Discussion

**FIG. 7.**
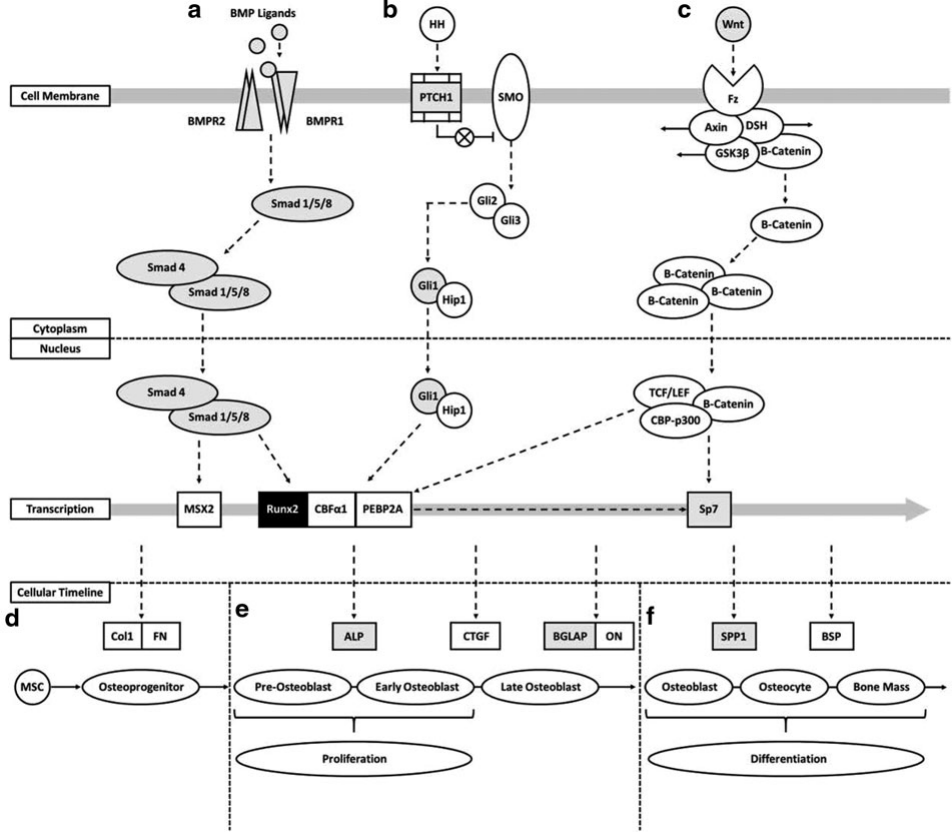
Pathway interaction schematic constructed to demonstrate overlap of gene expression data with critical osteogenic pathways. Pathways for BMP, HH, and Wnt signaling are shown with upregulated genes (gray) and the similarly expressed Runx2 (black) highlighted. **(a)** BMP ligands bind with BMP receptor subunits stimulating Smad 1/5/8 to couple with Smad 4. **(b)** HH binding to PTCH1 surface protein prevents inhibition of SMO, permitting production of Gli2/3 and subsequent transcription of Gli1 and Hip1. **(c)** Wnt interacting with Fz surface protein leads to release and subsequent accumulation of β-Cantenin, which then couples with TCF/LEF and CBP-p300. **(a–c)** BMP, HH, and Wnt pathways culminate in upregulation of crucial osteogenic transcription factors, including Runx2 and Sp7. **(d)** Naive MSCs are stimulated through matrix proteins toward osteolineage and differentiate to osteoprogenitor cells expressing primary cell attachment proteins, including Col1 and FN. **(e)** Induction of osteoprogenitor cells by osteo-related transcription factors results in early maturation stages for OB cells promoting proliferation and expression of ALP. Late-stage development of OB cells shows increased expression of osteocalcin (BGLAP) and ON proteins. **(f)** Mature OBs demonstrate reduced proliferative characteristics and enhanced expression of osteopontin (SPP1) protein. Matrix mineralization through OB activity during differentiation toward osteocytes results in bone mass formation. ALP, alkaline phosphatase protein; BMP, bone morphogenetic protein; Col1, collagen 1; FN, fibronectin; HH, Hedgehog; OB, osteoblastic; ON, osteonectin; SMO, smoothened.

As expected, based on previous *in vitro* and *in vivo* studies, the 3D nanocomposite scaffold composed of nHA/PU films interspersed with layers of DBPs was cytocompatible with adhMSCs. To elucidate the underlying mechanisms of this material, a combinatorial approach of transcriptomics and metabolomics was utilized. The scaffold demonstrated significant upregulation of genes closely associated with osteogenesis and indicated that a combined interaction of multiple pathways may be responsible for the osteobiologic characteristics exhibited by the material (Fig. 7). Specifically, the interaction of HH, Wnt, and BMP signaling pathways appears to play a crucial role in stimulating exposed cells.^18^

Members of the BMP family have been strongly correlated with osteogenesis and mineralization, in particular BMP-2/4/6/7, although recruitment of Smad 1/5/8 interacts with Smad 4 to regulate gene expression. Upregulation of BMP-2/4/6/7, BMP receptors, and Smad 1/4/5 may therefore provide evidence for activation of this pathway.^19,20^ In addition, coordination of this BMP signaling with both Wnt and HH signaling pathways is indicated by increased expression of essential pathway elements, including PTCH1, Gli1, and Wnt5A.^21^ HH, as well as Notch, signaling mechanisms have been previously demonstrated to have vital roles in bone remodeling and development through modulation of osteoblast and osteoclast activity.^22,23^

The described pathways culminate in upregulation of vital transcription factors, RUNX2 and Sp7, shown to elicit pro-osteogenic and antiadipogenic characteristics.^24,25^ This results in the enhanced production of key proteins for OB differentiation and ossification, including SPP1 and BGLAP, both of which were upregulated.^26^ As the expression of RUNX2 is similar between material-seeded cells and osteodifferentiated cultures, expression difference in other osteo-related targets may represent the mechanisms by which scaffold induces exposed cells toward an osteogenic lineage in 5 days.

Furthermore, hypoxia signaling and oxidative stress may also play important roles in facilitating the osteoinductive capacity of the scaffold, as these have been linked to skeletal development and bone promoting functions. Hypoxia signaling has been shown to have a role in the formation of endochondral bone, as well as the potential to modulate bone formation through manipulation of oxygen sensing,^27^ while oxidative stress, relating to an imbalance between generated radical oxygen species and available counteracting antioxidants, has demonstrated substantial influence on bone remodeling functions through suppression of osteoblast activity, temporarily reducing mineralization capacity and promoting resorption dynamics.^28^

These data combined with the upregulation of genes associated with cellular attachment functions, including cell–cell and cell–ECM adhesion/communication mechanisms, indicate that scaffold constructs facilitate cellular infiltration, attachment, and proliferation with subsequent stimulation of osteogenesis. By correlating the transcriptomic data with detected metabolite concentrations in cell-seeded scaffolds, fundamental pathways were constructed that appear to further support the function of scaffolds as osteogenic platforms.

Particularly the detection of ascorbate, a small molecule strongly associated with osteogenesis, in scaffold samples at markedly lower concentrations than those in scaffold blanks may indicate utilization of the metabolite by seeded cells. Xanthine concentration levels detected in cell-seeded scaffolds may also correlate to upregulation of oxidative stress pathway genes. Importantly, overlay of multiomic data through IMPaLA software revealed that overlapping regions between gene expression and metabolite concentrations were related to primarily cellular metabolism and signaling functions, which demonstrate that scaffolds are capable of facilitating cell–cell communication and supporting intricate intrastructural cell networks.

## Conclusions

As expected, the results of this study demonstrate that the scaffold material is both biocompatible and maintains osteogenic properties. Evaluation of the transcriptional landscape for scaffold exposed hMSCs compared with cells differentiated on polystyrene further indicated this osteogenic potential in the upregulation of expression in genes strongly associated with pro-osteogenic and cell attachment functions.

Fundamental pathway analysis of expression data revealed interactions among BMP, HH, and Wnt signaling mechanism as primary candidates for the osteobiologic characteristics of the material. Among these, HH appears to play a particularly crucial role and, thus, will be the target of future studies, which will implement HH-specific PCR profiler arrays (Qiagen) and inhibition assays to elucidate precise mechanisms.

The pro-osteogenic potential of the scaffold indicates that it may serve as an effective scaffold for both long and flat bone injuries, as it is capable of facilitating cellular in-growth and subsequent ossification. Further evaluation of scaffolds as delivery vehicles for drug and cell-based treatments is expected to yield enhanced osteobiologic graft treatments. Furthermore, application of similar experimental approaches for tissue samples from *in vivo* analyses of this material may provide invaluable insight as to its impact on native tissue, which is essential for translation to clinical applications.

## Supplementary Material

Supplemental data

Supplemental data

Supplemental data

Supplemental data

Supplemental data

Supplemental data

## Data Availability

The raw/processed data required to reproduce these findings cannot be shared at this time as the data also form part of an ongoing study.

## Abbreviations Used

adhMSC: adipose-derived human MSC
ALP: alkaline phosphatase protein
BMP: bone morphogenetic protein
cDNA: complimentary DNA
Col1: collagen 1
CPDB: ConsensusPathDB
DAVID: Database for Annotation, Visualization, and Integrated Discovery
DBP: decellularized bovine bone particle
DMEM-F12: Dulbecco's modified Eagle's medium-F12
ECM: extracellular matrix
EDTA: ethylenediaminetetraacetic acid
FN: fibronectin
HBSS: Hank's Balanced Salt Solution
HH: Hedgehog
IMPaLA: Integrated Molecular Pathway Level Analysis
KEGG: Kyoto Encyclopedia of Genes and Genomes
LC-MS: Liquid Chromatography-Mass Spectrometry
MoIs: metabolites of interest
mRNA: messenger RNA
MSC: mesenchymal stem cell
nHA: nano-hydroxyapatite
OB: osteoblastic
ON: osteonectin
PCR: polymerase chain reaction
PoIs: pathways of interest
PU: polyurethane
sPLS-DA: sparse partial least squares discriminant analysis
