# Supplementary material for: Multiomics Evaluation of Human Fat-Derived Mesenchymal Stem Cells on an Osteobiologic Nanocomposite
Source: Biores Open Access. 2020 Feb 21;9(1):37–50. doi: 10.1089/biores.2020.0005 (PMC7047255; doi:10.1089/biores.2020.0005)
Supplement: Supplemental data [file Supp_Table1.pdf]

Function	Gene	Fold Relation	Function	Gene	Fold Relation
Ossification	ACVR1	6.8474	Cartilage Condensation	BMPR1B	8.8697
	BGLAP	10.0949		COL2A1	99.1968
	BMP2	1508.4894		SOX9	63.509
	BMP3	80.3869	Osteoclast Differentiation	BGLAP	10.0949
	BMP4	11.7579		TNF	49.0287
	BMP6	4.0527		TNFSF11	118.7861
	BMPR1A	4.476	Osteoblast Differentiation	ACVR1	6.8474
	BMPR1B	8.8697		BGLAP	10.0949
	BMPR2	2.1222		BMP2	1508.4894
	CHRD	101.2812		BMP4	11.7579
	COL2A1	99.1968		BMP6	4.0527
	EGFR	2.9599		BMPR1A	4.476
	FGF2	4.7422		BMPR1B	8.8697
	FGFR2	4.8643		BMPR2	2.1222
	GDF10	4.0247		CHRD	101.2812
	GLI1	20.0967		FGF2	4.7422
	IGF1R	5.3848		FGFR2	4.8643
	MMP2	31.3898		GDF10	4.0247
	MMP8	24.7992		GLI1	20.0967
	MMP9	131.8014		NOG	3.2465
	NOG	3.2465		SMAD1	2.7363
	SMAD1	2.7363		SMAD3	11.2789
	SMAD3	11.2789		SP7	91.7025
	SOX9	63.509		SPP1	18.8813
	SP7	91.7025		TWIST1	5.4474
	SPP1	18.8813	Other Skeletal Development Genes	ALPL	3.3688
	TGFB1	13.6632		FGFR1	14.8826
	TGFB2	2.1819		TGFBR1	5.8383
	TNFSF11	118.7861			
	TWIST1	5.4474			
