# Supplementary material for: Multiomics Evaluation of Human Fat-Derived Mesenchymal Stem Cells on an Osteobiologic Nanocomposite
Source: Biores Open Access. 2020 Feb 21;9(1):37–50. doi: 10.1089/biores.2020.0005 (PMC7047255; doi:10.1089/biores.2020.0005)
Supplement: Supplemental data [file Supp_Table5.pdf]

Pathway Name	Overlapping Genes	Overlapping Metabolites
Signal Transduction	TGFB1; BMP2; FLT1; BMPR2; IGF1R; NOG; EGF; CALCR; PDGFA; EGFR; SPP1; NFKB1; ITGA2; MMP9; BMPR1A; SMAD1; SMAD2; SMAD3; SMAD4; SMAD5; VEGFA; VEGFB	N-Acetylglucosamine; UDP-N-acetylglucosamine; Dopamine; NAD+; Glutamate
Signaling by Receptor Tyrosine Kinases	PDGFA; EGFR; EGF; SPP1; FLT1; VEGFA; IGF1R; ITGA2; MMP9; VEGFB	NAD+
Signaling by TGF-beta family members	TGFB1; BMP2; SMAD2; BMPR1A; BMPR2; SMAD1; NOG; SMAD3; SMAD4; SMAD5	NAD+
Extracellular matrix organization	TGFB1; COL2A1; PDGFA; ITGAM; SPP1; BMP2; BMP4; ICAM1; ITGA2; MMP9	Ascorbate
Gene expression (Transcription)	TGFB1; EGFR; BMP2; SMAD3; SMAD1; SMAD3; BGLAP; SMAD4; SP7; VEGFA	Glutathione disulfide; Glutathione; Ascorbate; Glutamate; NAD+
Generic Transcription Pathway	TGFB1; EGFR; BMP2; SMAD3; SMAD1; SMAD3; BGLAP; SMAD4; SP7; VEGFA	Glutathione disulfide; Glutathione; Glutamate; NAD+
Metabolism of proteins	TGFB1; SPP1; BMP4; SMAD3; SMAD1; SMAD2; BGLAP; SMAD4; ALPL	Glucose 1-phosphate; Asparagine; Valine; Glutamate; NAD+; Inosine; N-Acetylglucosamine; UDP-N-acetylglucosamine
Post-translational protein modification	TGFB1; SPP1; BMP4; SMAD3; SMAD1; SMAD2; BGLAP; SMAD4; ALPL	N-Acetylglucosamine; UDP-N-acetylglucosamine; Glucose 1-phosphate; NAD+; Glutamate
Differentiation Pathway	TGFB1; PDGFA; BMP4; EGF; TNFSF11; NOG; VEGFA	Ascorbate
HIF-1 signaling pathway - Homo sapiens (human)	EGFR; FLT1; EGF; NFKB1; IGF1R; VEGFA	Ascorbate
Developmental Biology	SMAD3; ITGA2; SMAD4; EGFR; SMAD2	Glutamate
MAPK family signaling cascades	PDGFA; EGFR; EGF	Glutamate
MAPK1/MAPK3 signaling	PDGFA; EGFR; EGF	Glutamate
RAF/MAP kinase cascade	PDGFA; EGFR; EGF	Glutamate
Vitamin B12 Metabolism	NFKB1; ICAM1	Ascorbate; NAD+; Creatinine
Signaling by NOTCH	EGFR; EGF	N-Acetylglucosamine; UDP-N-acetylglucosamine
Collagen formation	COL2A1; MMP9	Ascorbate
Signaling by GPCR	EGFR; CALCR	Dopamine; NAD+; Glutamate
GPCR downstream signalling	EGFR; CALCR	Dopamine; Glutamate; NAD+
Cellular responses to external stimuli	NFKB1	Glutathione disulfide; Glutathione; NAD+
Cellular responses to stress	NFKB1	Glutathione disulfide; Glutathione; NAD+
cAMP signaling pathway - Homo sapiens (human)	NFKB1	Dopamine
Collagen biosynthesis and modifying enzymes	COL2A1	Ascorbate
Protein digestion and absorption - Homo sapiens (human)	COL2A1	Asparagine; Valine; Glutamate
