# Supplementary material for: Multiomics Evaluation of Human Fat-Derived Mesenchymal Stem Cells on an Osteobiologic Nanocomposite
Source: Biores Open Access. 2020 Feb 21;9(1):37–50. doi: 10.1089/biores.2020.0005 (PMC7047255; doi:10.1089/biores.2020.0005)
Supplement: Supplemental data [file Supp_Table6.pdf]

Pathway	Overlapping Genes	p-value	Benjamini
TGF-beta Signaling Pathway	18	1.10E-17	1.60E-15
Cellular Response to BMP Stimulus	11	2.80E-16	1.20E-13
Positive Regulation of Pathway-restricted SMAD Protein Phosphorylation	12	9.30E-16	2.70E-13
BMP Signaling Pathway	13	5.40E-15	1.10E-12
Signaling Pathways Regulating Pluripotency of Stem Cells	18	9.10E-14	4.30E-12
Positive Regulation of Bone Mineralization	9	9.50E-12	1.00E-09
Positive Regulation of Osteoblast Differentiation	10	2.60E-11	2.30E-09
Extracellular Space	41	2.80E-22	4.10E-20
SMAD Protein Signal Transduction	12	2.00E-11	2.80E-12
Negative Regulation of Canonical Wnt Signaling Pathway	5	1.10E-02	9.70E-02
Growth Factor	14	1.40E-14	7.50E-13
Osteogenesis	5	1.90E-05	2.20E-04
Positive Regulation of Ossification	4	3.10E-05	6.20E-04
Osteoclast Differentiation	10	1.20E-05	1.20E-04
FoxO Signaling Pathway	10	1.50E-05	1.30E-04
Adherens Junction	7	1.20E-04	7.20E-04
Cell-cell Junction Organization	3	4.00E-03	4.10E-02
Angiogenesis	11	2.80E-07	9.00E-06
