# Supplementary material for: Multiomics Evaluation of Human Fat-Derived Mesenchymal Stem Cells on an Osteobiologic Nanocomposite
Source: Biores Open Access. 2020 Feb 21;9(1):37–50. doi: 10.1089/biores.2020.0005 (PMC7047255; doi:10.1089/biores.2020.0005)
Supplement: Supplemental data [file Supp_Table2.pdf]

Function	Gene	Fold Relation
Bone Mineralization	ACVR1	6.8474
	BGLAP	10.0949
	BMP2	1508.4894
	BMP4	11.7579
	BMP6	4.0527
	BMPR1A	4.476
	BMPR1B	8.8697
	BMPR2	2.1222
	FGFR2	4.8643
	SMAD3	11.2789
	SOX9	63.509
	TGFB1	13.6632
	TWIST1	5.4474
Calcium Ion Binding & Homeostasis	BGLAP	10.0949
	CALCR	12.8073
	EGF	37.0711
	FGF2	4.7422
	MMP2	31.3898
	MMP8	24.7992
	TGFB1	13.6632
	VDR	2.0217
