# Supplementary material for: Multiomics Evaluation of Human Fat-Derived Mesenchymal Stem Cells on an Osteobiologic Nanocomposite
Source: Biores Open Access. 2020 Feb 21;9(1):37–50. doi: 10.1089/biores.2020.0005 (PMC7047255; doi:10.1089/biores.2020.0005)
Supplement: Supplemental data [file Supp_Table3.pdf]

Function	Gene	Fold Relation
Cell-Cell Adhesion	BMPR1B	8.8697
	COL14A1	5.4348
	COL2A1	99.1968
	EGFR	2.9599
	ICAM1	4.4042
	SOX9	63.509
	TGFB1	13.6632
	TNF	49.0287
	TNFSF11	118.7861
Cell-ECM Adhesion	CD36	3.8519
	COL2A1	99.1968
	ITGA2	42.0944
	ITGAM	29.4233
	SMAD3	11.2789
Other Cell Adhesion Molecules	BGLAP	10.0949
	COL15A1	6.2285
	TNF	49.0287
