# Supplementary material for: Multiomics Evaluation of Human Fat-Derived Mesenchymal Stem Cells on an Osteobiologic Nanocomposite
Source: Biores Open Access. 2020 Feb 21;9(1):37–50. doi: 10.1089/biores.2020.0005 (PMC7047255; doi:10.1089/biores.2020.0005)
Supplement: Supplemental data [file Supp_Table4.pdf]

Function	Gene	Fold Relation	Function	Gene	Fold Relation
Hedgehog Signaling	BCL2	42.7478	TGFβ Signaling	IFRD1	4.8605
	BMP2	802.1775		MYC	4.9398
	BMP4	6.267	WNT Signaling	FOSL1	7.0508
	PTCH1	23.3353		CCND2	2.2519
	WNT1	178.2524		MMP7	3.1335
	WNT2B	2.709		MYC	4.9398
	WNT3A	127.508	NFκB	BCL2A1	137.9286
	WNT5A	2.3205		BIRC3	3.6921
	WNT6	254.4276		CCL5	8.9039
Oxidative Stress	FTH1	5.3435		ICAM1	2.1452
	GCLC	2.5867		IFNG	119.5202
	GCLM	16.3866		TNF	68.9643
	GSR	3.1191	Notch Signaling	HES1	4.0309
	HMOX1	778.4413		HES5	149.8918
	SQSTM1	47.6513		HEY1	8.3462
	TXN	2.3583		HEY2	63.9015
	TXNRD1	4.4417		HEYL	78.672
Hypoxia Signaling	CA9	39.6096		LFNG	13.7794
	EPO	76.8751		NOTCH1	12.1631
	HMOX1	778.4413			
	SLC2A1	2.1852			
	VEGFA	2.1452			
